# Association rule mining with a special rule coding and dynamic genetic algorithm for air quality impact factors in Beijing, China

**DOI:** 10.1371/journal.pone.0299865

**Published:** 2024-03-04

**Authors:** Xiaoxuan Wu, Qiang Wen, Jun Zhu

**Affiliations:** 1 School of Artificial Intelligence and Big Data, Hefei University, Hefei, Anhui, China; 2 Key Laboratory of Intelligent Building and Building Energy Efficiency, Anhui Jianzhu University, Hefei, Anhui, China; Alagappa University, VIET NAM

## Abstract

Understanding air quality requires a comprehensive understanding of its various factors. Most of the association rule techniques focuses on high frequency terms, ignoring the potential importance of low- frequency terms and causing unnecessary storage space waste. Therefore, a dynamic genetic association rule mining algorithm is proposed in this paper, which combines the improved dynamic genetic algorithm with the association rule mining algorithm to realize the importance mining of low- frequency terms. Firstly, in the chromosome coding phase of genetic algorithm, an innovative multi-information coding strategy is proposed, which selectively stores similar values of different levels in one storage unit. It avoids storing all the values at once and facilitates efficient mining of valid rules later. Secondly, by weighting the evaluation indicators such as support, confidence and promotion in association rule mining, a new evaluation index is formed, avoiding the need to set a minimum threshold for high-interest rules. Finally, in order to improve the mining performance of the rules, the dynamic crossover rate and mutation rate are set to improve the search efficiency of the algorithm. In the experimental stage, this paper adopts the 2016 annual air quality data set of Beijing to verify the effectiveness of the unit point multi-information coding strategy in reducing the rule storage air, the effectiveness of mining the rules formed by the low frequency item set, and the effectiveness of combining the rule mining algorithm with the swarm intelligence optimization algorithm in terms of search time and convergence. In the experimental stage, this paper adopts the 2016 annual air quality data set of Beijing to verify the effectiveness of the above three aspects. The unit point multi-information coding strategy reduced the rule space storage consumption by 50%, the new evaluation index can mine more interesting rules whose interest level can be up to 90%, while mining the rules formed by the lower frequency terms, and in terms of search time, we reduced it about 20% compared with some meta-heuristic algorithms, while improving convergence.

## Introduction

In recent years, environmental pollution has emerged as a critical concern, with air pollution gaining increasing prominence. Pollution arises from numerous sources, including non-compliant factory emissions, vehicle exhaust emission, construction-related dust, and agricultural practices such as straw burning. These unscientific activities lead to abnormal changes in the concentration of carbon, nitrogen, sulfur, particulate matter with diameters less than 2.5 micrometers (PM2.5) and 10 micrometers (PM10), as well as O_3_ in the air, resulting in atmospheric pollution. Air Quality Index (AQI) serves as a metric for ranking air pollution levels, influenced by a myriad of factors. These encompass meteorological elements (cloud cover, sunlight, precipitation, wind speed and direction) and geographical variables (altitude, latitude, and longitude). Previous studies have typically associated AQI categories with individual influencing factors, determining whether the category is positively, negatively, or not correlated with the given factor. This study aims to uncover the correlation between AQI and multiple factors within a specific category, as well as the degree of this correlation. Furthermore, it seeks to explore the correlation between certain factors under the sample data. The derived rules can serve as guiding principles for air quality improvement. For instance, mining from the sample data reveals that the air quality within the second category, denoted as ’good’, is influenced by the concentration of SO_2_ in the second gradient and NO_2_ concentration within the first gradient interval range.

Association rule mining (ARM) [1] is an unsupervised learning technique in the field of data mining, which is used to mine rules from a specific scenario, research or transaction process database. Its original purpose was to provide interesting relationships, associations, or frequent patterns between sets of items in database transactions. Intriguingly, ARM’s genesis can be traced back to an observed co-purchasing trend of beer and diapers in the renowned Walmart supermarket chain. Today, ARM finds applications across diverse sectors. In retail, it informs product placement strategies to optimize promotions; in medicine, it helps uncover relationships between ailments and treatment strategies. For example, Elif et al. used numerical ARM to identify potentially important rules between Parkinson’s disease and voice change characteristics [2, 3]; in recommendation engines, it facilitates matching users based on shared preferences; and in safety research, ARM has been instrumental in identifying principal causative factors behind accidents [4–6]. In mechanical fault diagnostics, ARM facilitates rapid fault source identification, enabling immediate remediation [7]. Considering the above broad application scenarios, this study employs ARM to elucidate correlations among influencing factors in air quality, particularly within the Beijing region of China. However, several challenges need to be overcome before successful application in finding associations between air quality factors:

·Traditional ARM is carried out on the basis of discrete data types. Since some influencing factors of air quality are continuous, it is difficult to mine rules in the case of pre-processing and integration of different types of data.

·Most of the previous ARM techniques were designed to filter out items with a certain frequency by artificially setting the support degree, and then explore their relationships, which made it challenging to find the relationship between items with low frequency. Such an operation can undervalue the significance of anomalies in air quality datasets, resulting in biased interpretations of factors impacting air quality.

·The performance of ARM combined with other algorithms still has room for improvement. For example, in the aspect of space storage, the traditional technologies, including the Apriori algorithm, demand multiple database traversals when discerning item set associations and store each item contained in the database transaction with one unit storage space, which yield candidate item sets at exponential magnitudes and wastes storage space. In the mining of rules, the quality and time efficiency of rules can still be improved.

### Contribution of this paper

To solve these problems, a novel genetic algorithm-Dynamic Genetic Algorithm (DGA) is introduced in this paper. The key contributions of this study are as follows:

1. The manuscript proposes the concept of dynamic threshold of mutation rate and crossover rate, which improves its ability to accurately locate the optimal solution, thus minimizing the convergence time.
2. A well-conceived encoding strategy aligns seamlessly with the research objectives, as reflected in the efficient complexity of the Dynamic Genetic Association Rule Mining (DGAARM) algorithm.
3. The weighted optimization of the overall evaluation index can ensure that more valuable rules can be extracted.

### Related work

Agrawal et al. [8] first introduced the concept of ARM which provides an opportunity to discover item-to-item relationships from a data set containing a large number of variables [9]. Subsequently, Apriori algorithm [10] was proposed and received wide attention. Despite its broad attention, the Apriori algorithm, in its quest for frequent itemsets, produces an excessive number of candidate items. This led to innovations like the approach by Han et al. [11], who employed a tree data structure to organize transaction data and conducted depth-first traversal, resulting in the renowned Frequent Pattern Growth (FP-Growth) algorithm. However, this requires preliminary tasks such as item frequency ranking and the laborious construction of a conditional Frequent Pattern Tree (FP-tree). The emergence of Eclat algorithm changes the horizontal representation of data to vertical representation, and turns multiple scanning of database for Apriori and FP-Growth to only two times when calculating item set support, which is completed by intersecting Tidset after vertical representation of data. However, because Eclat algorithm takes a long time to find the intersection when there is a large amount of data, Zhang et al. [12] used minwise hashing and estimator to quickly calculate the intersection size of multiple item sets, thus improving its efficiency. Based on the fact that Eclat consumes a large amount of memory space and computation time in data reading and computation under a large amount of data, and generates a large amount of redundant data in the process, Wang et al. [13] improved the pruning strategy of Eclat, which reduced the generation of redundant frequent items, and improved the efficiency of the algorithm. In recent years, many researchers have improved on the basis of the above two algorithms. For example, in the analysis of tower crane accidents, Liu et al. [14] introduced the interest degree (I) model with upper and lower bound idea, lifting degree and leverage ratio evaluation indexes based on Apriori algorithm, which reduced the number of redundant rules, but did not improve the algorithm performance. Given the large memory occupied by the FP-Growth algorithm to construct the pattern tree by using the entire transaction database, the low operation efficiency of the algorithm, and the poor timeliness of data mining, Yu and Liu et al. [15] proposed the MFP-tree algorithm. When traversing the transaction database for the first time, the algorithm calculates the support degree of all items, deletes the items that do not meet the threshold according to the artificially set support threshold, re-sorts the items of each transaction, constructs a database subset of each item according to the frequent 1-item set, and then carries on the FP-Growth algorithm on this subset. Experiments show that MFP-tree algorithm has certain advantages over FP-Growth algorithm when mining database is larger or constraint conditions are strict.

Furthermore, many ARM applications rely heavily on support and confidence degree thresholds during extraction mining. For instance, Wang et al. [16] applied a support degree of 0.2 using the MapReduce model to enhance the Apriori algorithm, while Liu et al. [17] utilized a confidence threshold of 0.5 with the parallel FP-Growth algorithm to decipher rules between temperature and salinity in marine Argo datasets. This methodology can inadvertently filter out items and rules below set thresholds, however sometimes infrequent items and rules are of interest to researchers instead.

The meta-heuristic algorithm based on the improvement of heuristic algorithm includes Genetic Algorithm (GA) and swarm intelligence algorithms such as Particle Swarm Optimization (PSO) algorithm and whale optimization algorithm (WOA). Among them, GA encompasses many variants such as classical, parallel, hierarchical, adaptive, and hybrid algorithms [18–21], and showed good performances for optimization problems. Since ARM is widely used in various fields for knowledge discovery or pattern association, some researchers combine heuristic algorithms with association rules to improve the time performance and result optimization of algorithms. For example, S. Sharmila et al. [22] combines WOA with fuzzy logic to identify frequent items and generate association rules. In the study of numerical association rules, Elif et al. [23] proposes a new hybrid multi-objective evolutionary optimization algorithm based on differential evolution (DE) and sine and cosine algorithm. The sine-cosine algorithm can effectively prevent premature convergence and stagnation in the iterative process, and improve the overall search ability and convergence performance of the algorithm. Given ARM only considers the frequency of items in the item set to find the item set of interest, which cannot reflect the usefulness or preference of users to quantify products with different values, Kannimuthu et al. [24] introduced a high-utility itemset mining algorithm. Adopting GA to optimize the PSO algorithm to avoid the combination explosion problem and the problem of early stagnation of algorithm search, it turned out that the number of candidate item sets is reduced effectively and the convergence performance of the algorithm is improved. In order to avoid the combination explosion problem in the study of web service composition, S. Kannimuthu et al. [25] proposed a hybrid genetic algorithm (HGA), which combines quantum operators and classical genetic operators, to mine efficient web service composition. The chromosome constructed by superposition qubits based on quantum computing model achieves good results in terms of running time and memory consumption. In addition, relevant researchers regard the support, confidence and other evaluation indicators in ARM as multiple objectives and adopt multi-objective optimization association rule mining. For example, Tyagi et al. [26] extracted valuable rules by multi-objective particle swarm optimization (MOPSO) in the collaborative filtering of recommendation system to improve the recommendation quality. In addition, since users have prior knowledge and research trends of some key items in practical applications, association rules containing key items are more valuable and meaningful for these users. Therefore, Hu et al. [27] proposed the Animal Dynamic Migration Optimization (ADMO) algorithm for directional mining rules. By changing the constant direction migration of animals in the original animal migration algorithm to the dynamic direction correction mode, good results are obtained in key rules, rule optimization, memory consumption and execution time.

AQI is a gauge of daily air quality, segmented into six categories from Class I to Class VI. Each AQI level has distinct implications for human health, influencing t well-being and societal progress. Initiatives to understand the determinants of air quality, diminish pollution sources, and thwart the interplay of multiple pollutants are pivotal for air quality enhancement. Current research has made significant strides in deciphering the factors influencing air quality. For example, Li et al. [28] undertook linear correlation and multiple regression analyses on monthly air quality variations and meteorological elements across cities. The meta-analysis based on correlation and regression coefficients showed the relationship between certain pollution factors and meteorological variables. Notably, PM2.5 concentrations showed correlation with all meteorological metrics, except wind speed. In contrast, PM10 and O_3_ concentrations exhibited links with all meteorological variables; however, O3’s correlation direction with meteorological indicators deviated from that of PM2.5 and PM10. Zhu [29] delved into the spatiotemporal and socio-economic attributes of regional air pollution by devising a panel data gray correlation clustering model and a gray entropy test model. Duan et al. [30] used GA to optimize subregion-level priority of precursor emission reductions and combined Self-Organizing Map (SOM) and WRF-CAMx for the collaborative control of PM2.5 and O_3_ in Beijing-Tianjin-Hebei and the surrounding area (BTHSA, “2 + 26” cities).

### Organization of this paper

The structure of this paper is as follows: The Materials and methods section introduces the general framework, data pre-processing of the air quality data set used, the basic concept of ARM and DGAARM algorithm. The Result and discussion section introduces the experimental results and related discussions. Finally, a summary of the work in the Conclusions section of this paper is presented.

## Materials and methods

### General framework

**Fig 1:**
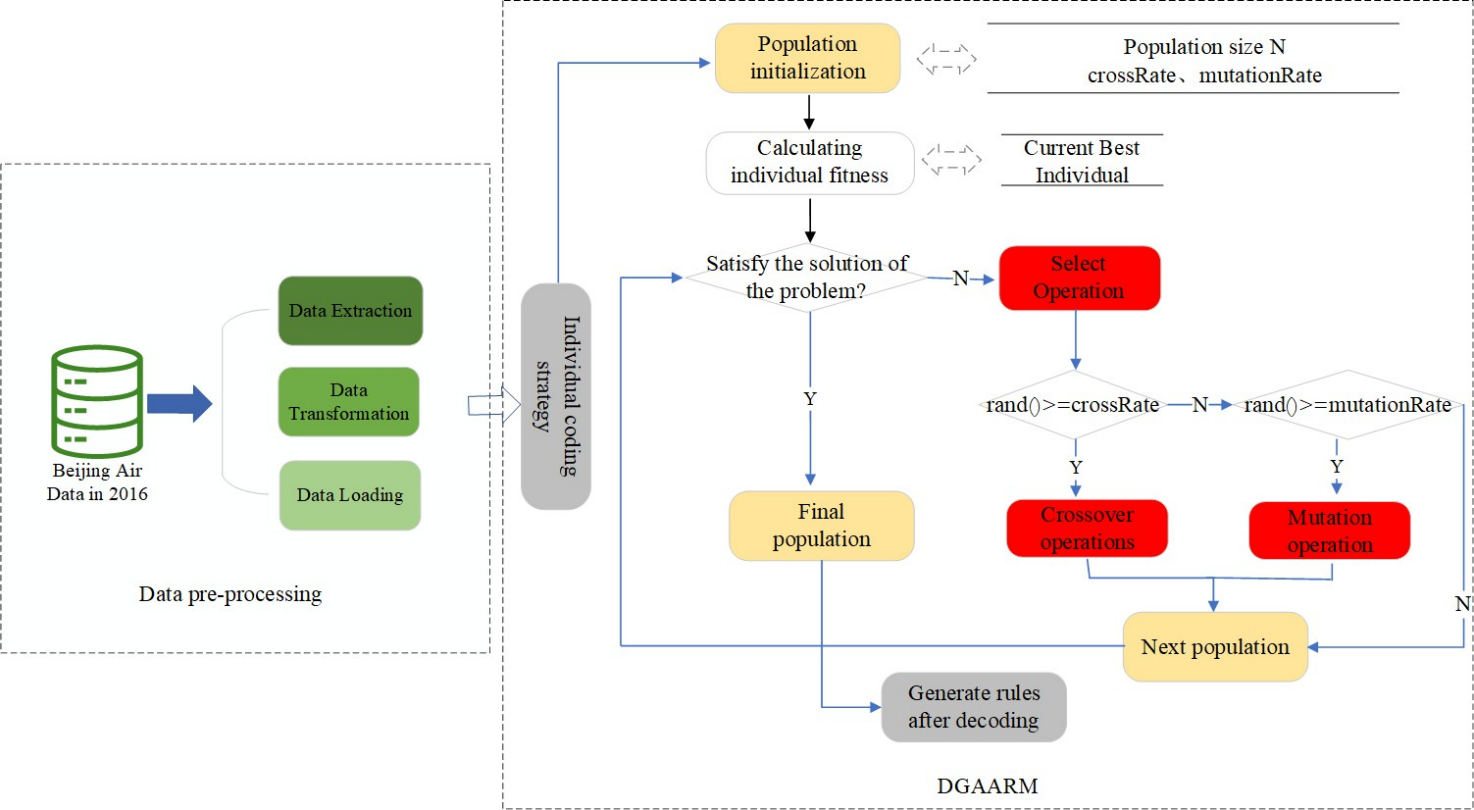
General framework for mining association rules of air quality impact factors based on DGAARM.

In this study, we apply the DGAARM algorithm to optimize the performance of ARM. We realize the mining of interesting air quality association rules without artificially setting minimum support threshold, and design a unique coding method to optimize the spatial storage of the rules, and optimize the convergence performance by combining dynamic crossover and variation rate in the mining process. Initially, we pre-process the 2016 annual air data from Beijing, which involves data extraction, transformation, and loading. Subsequently, we target and code the chromosome genes in accordance with the problem’s specificity. We then employ the DGAARM algorithm to unearth the rules governing air quality influencing factors, before comparing its performance with other classical association rule algorithms. The overarching framework of the proposed method is depicted in Fig 1.

### Data pre-processing

The data pre-processing stage is divided into three parts: firstly, Data Extraction, then, Data Transformation, lastly, Data Loading, as shown in the left part of Fig 1. In the data extraction phase, the experimental dataset is utilized in this study which was obtained from the environmental cloud of Nanjing Yunchuang Big Data Technology Co., LTD. (Nanjing, China). We accessed hourly meteorological records data and hourly air quality monitoring data for Beijing, spanning from 1 January 2016 to 31 December 2016, which were recorded by 12 monitoring sites. We spliced two parts of the data and selected one of the sites with a total of 8784 records. We loaded these data into the Pandas library’s DataFrame object for feature extraction. The properties of the raw data are detailed in Table 1.

**Table 1:** The raw data description table.

Statistical name	Statistical parameters
Traits	TIME, CITY CODE, Weather conditions, Temperature, Body temperature, Barometric pressure, Relative humidity, Rainfall, Wind direction, Wind speed, Air quality index, PM_2.5_ concentration, PM_10_ concentration, NO_2_ concentration, SO_2_ concentration, O_3_ concentration, CO concentration
Number of features	18
Number of data bars	8784

Then irrelevant attributes such as time, city-specific invariant attributes (given the city is consistently Beijing), and body temperature were removed. Subsequently, the remaining features were numerically assessed, leading to the deletion of non-numeric data types. The results of the post-extraction are detailed in Table 2. In the data transformation phase, the data were discretized, and the results are shown in Table 3. Weather conditions were manually discretized, while the final seven features were categorized based on the Ambient Air Quality Standard (GB3095-2012) and AQI Technical Provisions (for Trial Implementation) HJ633-2012. The dataset contains weather categories such as "sunny" and "haze", among 16 other classification categories. Temperature data, ranging from -15.1°C to 37.3°C, was organized into 17 classes including descriptors like "deep chill" and "the Great Cold". Air pressure is classified using neighboring relative air pressure values: values below one standard atmosphere are deemed "low pressure" and those above as "high pressure". Relative humidity classifications are "dry" for values below 30%, "humid" for above 80%, and "normal" for the intermediate range. Rainfall data, with a range from 0mm to 32.5mm, is categorized into four levels: R0 to R3. These classifications stem directly from the specific range of each feature’s data. Finally, load the final data into the DGAARM algorithm model, completing the classical ETL (Extract, Transform, Load) processes of data loading, feature extraction, and data transformation.

**Table 2:** Data description table after ETL operation.

Statistical name	Statistical parameters
Traits	Weather conditions, Temperature, Barometric pressure, Relative humidity, Rainfall, Wind direction, Wind speed, Air quality index, PM_2.5_ concentration, PM_10_ concentration, NO_2_ concentration, SO_2_ concentration, O_3_ concentration, CO concentration
Number of features	14
Number of data bars	6430

**Table 3:** Characteristics description table after discretizing operation.

Feature Name	Values after discretizing operation
Weather conditions	Sunny, Hazy, Cloudy, Overcast, Light rain, Moderate to heavy rain, Heavy rain, Rain showers, Thunderstorms, Fog, Sleet, Light snow, Moderate to heavy snow, Floating layer, Moderate rain, Rainstorm.
Temperature	Deep Chill (-15~20°C), The Great Cold (-10~15°C), Little cold (-10~-5°C), Lightly cold (-5~0°C), Slightly cold (0~5°C), Cool (5~10°C), Warm and cool(10~12°C), Slightly warm and cool (12~14°C), Mild (14~16°C), Slightly mild (16~18°C), Warm (18~20°C), Warm but not hot (20~22°C), Slightly hot (22~25°C), Hot (25~28°C), Summer hot (28~30°C), Very hot (30~35°C), Extremely hot (35~39°C).
Barometric pressure	Low-pressure, High pressure.
Relative humidity	Dry (<30%), Normal (30%~80%), Humidity (>80%).
Rainfall	R0 (< = 5mm), R1 (5~15mm), R2 (15~30mm), R3 (30~70mm).
Wind direction	East, Southeast, South, Southwest, West, Northwest, North, Northeast, No sustained wind direction.
Wind power	Light breeze, Force3 wind, Force4 wind, Force5 wind.
Air quality index	AQI-1 ~ AQI-6^a^
PM_2.5_ concentration	PM2_5_I ~ PM2_5_VII^b^
PM_10_ concentration	PM10_I ~ PM10_VIII^c^
NO_2_ concentration	NO_2__I ~ NO_2__IV^d^
SO_2_ concentration	SO_2__I ~ SO_2__III^e^
O_3_ concentration	O_3__I ~ O_3__V^f^
CO concentration	CO_I ~ CO_III^g^

^a^AQI has been classified as AQI-I to AQI-VI, representing the first rank to the sixth rank of air quality rank which ranges from 0 to 50, 51 to 100, 101 to 150, 151 to 200, 201 to 300, and more than 300, respectively.

^b^PM2.5 has been classified as PM2_5_I to PM2_5_VII, which range from 0 to 35μg/m^3^, 35 to 75μg/m^3^, 75 to 115μg/m^3^, 115 to 150μg/m^3^, and 150 to 250μg/m^3^, respectively.

^c^PM10 has been classified as PM10_I to PM10_VIII, which range from 0 to 50μg/m^3^, 50 to 150μg/m^3^, 150 to 250μg/m^3^, 250 to 350μg/m^3^, 350 to 420μg/m^3^, 420 to 500μg/m^3^, 500 to 600μg/m^3^and more than 600μg/m^3^, respectively.

^d^NO2 has been classified as NO2_I to NO2_V, which range from 0 to 40μg/m^3^, 40 to 80μg/m^3^, 80 to 180μg/m^3^, 180 to 280μg/m^3^and more than 280μg/m^3^, respectively.

^e^SO2 has been classified as SO2_I to SO2_III, which range from 0 to 50μg/m^3^, 50 to 150μg/m^3^ and more than 150μg/m^3^, respectively.

^f^O3 has been classified as O3_I to O3_V, which range from 0 to 100μg/m^3^, 100 to 160μg/m^3^, 160 to 2150μg/m^3^, 215 to 265μg/m^3^ and more than 265μg/m^3^, respectively.

^g^CO has been classified as CO_I to CO_III, which range from 0 to 2 mg/m^3^, 2 to 4 mg/m^3^and more than 4 mg/m^3^, respectively.

### ARM

The algorithm for ARM is primarily concerned with identifying patterns in the form of X = >Y within a database, where X and Y are mutually exclusive sets. The process of ARM is bifurcated into two stages: the extraction of frequent item sets and the subsequent discovery of association rules. The initial stage is characterized by the use of support (sup), while the latter stage employs evaluation metrics such as confidence (conf) and lift (lift). Both support and confidence serve as indicators of the robustness of the association rules [31].

#### Definition 1. Association rules

Consider a transaction database D, where each distinct attribute is represented as a unique item i. This results in an itemset $I=\{i_{1},i_{2},i_{3},\ldots ,i_{n_{1}}\}$, where n_1_ signifies the total number of attributes in the database. Let $T=\{t_{1},t_{2},t_{3},\ldots ,t_{n_{2}}\}$ represent the set of transactions, with n_2_ indicating the overall count of transactions within the database. The association rule takes the form $\left\{i_{x_{1}},i_{x_{2}},i_{x_{3}},\ldots ,i_{x_{k}}\}\Rightarrow \{i_{s_{1}},i_{s_{2}},i_{s_{3}},\ldots ,i_{s_{k}}\right\}$, where $x_{1},x_{2},x_{3},\ldots ,x_{k},s_{1},s_{2},s_{3},\ldots ,s_{k}\in [1,2,3,\ldots ,n_{1}]$, $\{i_{x_{1}},i_{x_{2}},i_{x_{3}},\ldots ,i_{x_{k}}\}\cap \{i_{s_{1}},i_{s_{2}},i_{s_{3}},\ldots ,i_{s_{k}}\}=\emptyset$. The left-hand side of the symbol = > is commonly known as the antecedent, while the right-hand side is referred to as the consequent.

#### Definition 2. Support [32]

Support refers to the proportion of transactions that contain a specific itemset, as determined by ∂(*X*), relative to the total number of transactions within the database. This is computed using Eq (1).


$$sup(X)=\frac{\partial (X)}{\left|D\right|}$$ (1)

#### Definition 3. Confidence [33]

Confidence serves as a metric quantifying the strength of association between the antecedent and the consequent of a rule. A higher confidence value signifies a stronger association between the antecedent and the consequent. It is computed using Eq (2).


$$\mathrm{conf}(X\to Y)=\frac{\partial (X\cup Y)}{\partial (X)}$$ (2)

Wherein Y is also a subset of the item set I. In this context, X represents the antecedent and Y signifies the consequent, with *X*∪*Y* denoting the set encompassing all items of the rule. The confidence of the rule is determined by calculating the ratio of the number of transactions in the database that include all items of the rule to the number of transactions that contain all items in the antecedent of the rule. Consequently, this ratio represents the probability of Y’s occurrence given the occurrence of X.

#### Definition 4. Lift

Lift serves as an indicator of the extent to which the presence of one item influences the likelihood of another item’s occurrence. It provides insight into the correlation between items, whether positive, negative, or non-existent. It is computed using Eq (3).


$$lift(X\to Y)=\frac{conf(X\to Y)}{\sup(Y)}=\frac{\sup(X\to Y)}{\sup(X)\cdot \sup(Y)}$$ (3)

A positive correlation exists between X and Y when *lift*(*X*→*Y*)>1, while a negative correlation is observed when *lift*(*X*→*Y*)<1. In instances where *lift*(*X*→*Y*)≡1, X and Y are deemed to be independent, indicating no correlation.

### DGAARM algorithm

The DGAARM algorithm proposed in this paper integrates genetic algorithm into ARM to quickly reveal the rule between various air quality factors and Air quality index (AQI) in a specific environment. DGAARM algorithm consists of four key parts: chromosome gene coding, chromosome population initialization, selection during algorithm execution, crossover and mutation operators design, and chromosome population renewal iteration process. By introducing multi-information unit points, dynamic crossover rate and dynamic mutation rate, the optimal solution discovery ability of genetic algorithm is enhanced.

#### Coding design of genes

The encoding phase of DGAARM focuses on representing association rules in binary codes. Two common methods are the Pittsburgh method, which uses a single chromosome to describe ’n’ association rules, and the Michigan method, which uses one chromosome for each association rule. In this study, the latter approach was employed for chromosome design.

**Fig 2:**
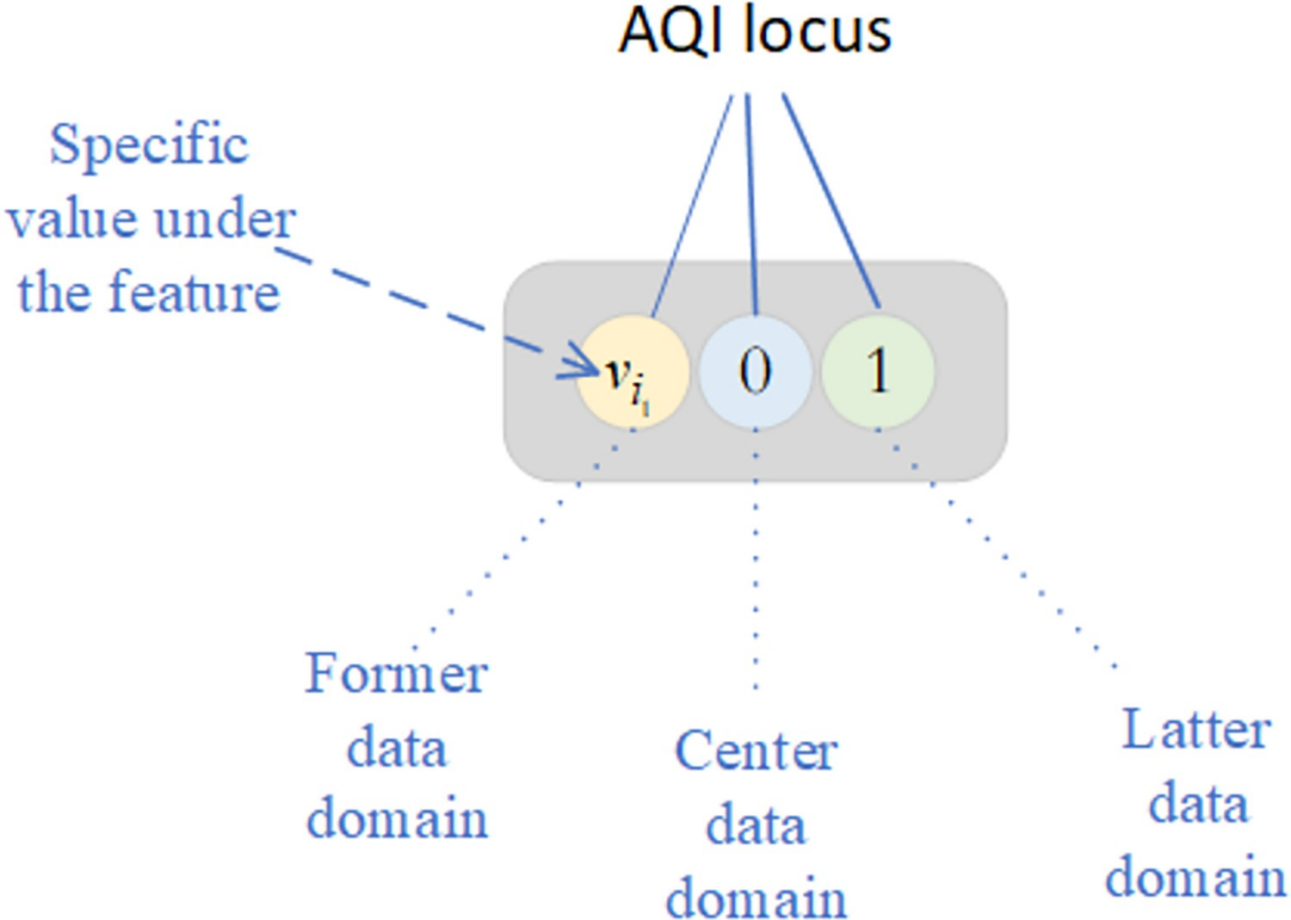
Gene loci under the feature of AQI.

**Fig 3:**
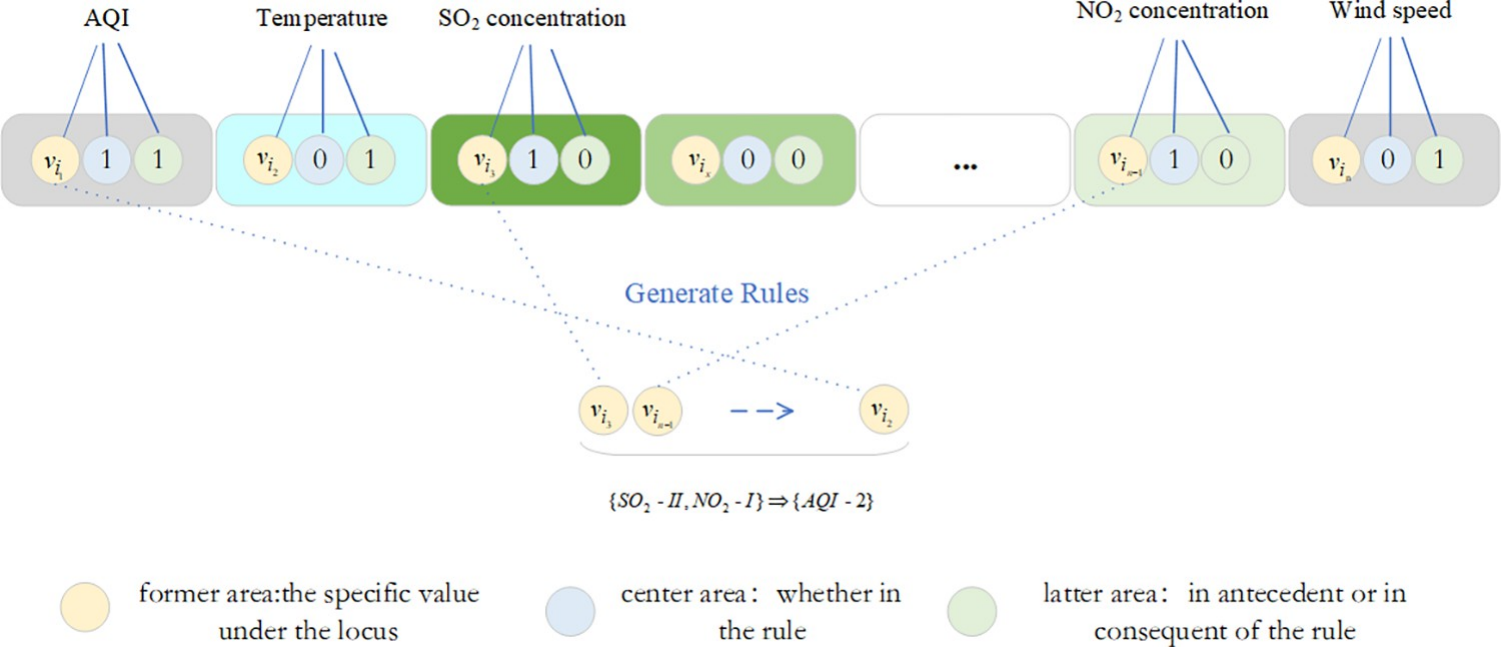
The generated rule case diagram.

The number of loci in a chromosome is dictated by the transaction database features. In this experiment, 14 features resulted in 14 loci, each encapsulated by the ATGC class, subdivided into the former, center, and latter data domains. The former domain stores a specific discrete category under a feature; the center domain indicates the presence (1) or absence (0) of the item set stored by the former domain in the rule; the latter domain indicates whether the item set is in the predecessor (0) or the posterior (1) of the rule. A representation of the gene locus for air quality characteristics and the rule encoding of $\left\{{\mathrm{SO}}_{2}-\mathrm{II},{\mathrm{NO}}_{2}-I\}\Rightarrow \{AQI-II\right\}$ is provided in Figs 2 and 3, respectively.

#### Initialization of primordial chromosome population

Each chromosome in the population carries a wealth of information within its genes, marked by random numbers assigned to each domain of every locus. The range of these random numbers varies across domains; in the former domain, the range corresponds to the discrete category count under the feature of that locus, whereas in the center and latter domains, the range is confined to the set {0,1}.

After encapsulating the former, center, and latter domains into a gene, the validity of the newly generated chromosome is verified using the JudgeGene function. This process scrutinizes whether the front and rear sections of the chromosome are populated and checks the chromosome’s existence within the dataset. Should these conditions not be met, the chromosome is regenerated.

**Fig 4:**
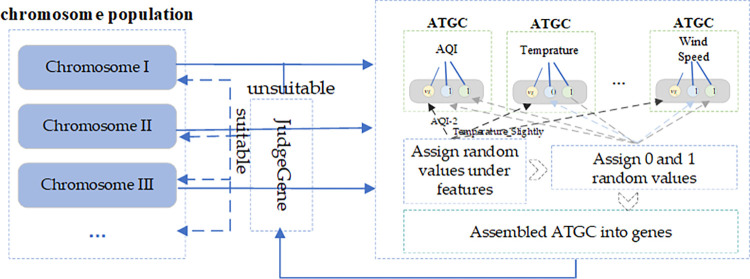
Initialization process of primordial chromosome population.

The specific algorithmic process can be outlined as follows and is illustrated in Fig 4:

- For each chromosome in the population, each locus’s former, center, and latter domains are initialized according to the discrete label;
- Once all loci are initialized, they are assembled into genes;
- The JudgeGene function then assesses the gene’s rationale. If deemed suitable, the next chromosome’s initialization is performed, and so forth until the entire chromosome population is processed. Should the gene fail the check, the chromosome is re-initialized. Taking chromosome I as an example, it is properly initialized because the center area at all loci is not all 0 and the latter area is all 0 or all 1.

#### Chromosome selection, crossover, mutation

In this study, we introduce the DGAARM, which incorporates a unique roulette wheel-based strategy in Genetic Algorithm (GA). Unlike traditional approaches, our strategy favors chromosomes with smaller fitness values, as determined by Eq (4).


$$fitness=w_{1}*\sup(X\to Y)+w_{2}*conf(X\to Y)+w_{3}*lift(X\to Y)$$ (4)

In this context, w_1_, w_2_, and w_3_ denote weights such that their sum equals one. Chromosomes with smaller fitness values possess a higher probability of undergoing subsequent crossover and mutation operations.

**Fig 5:**
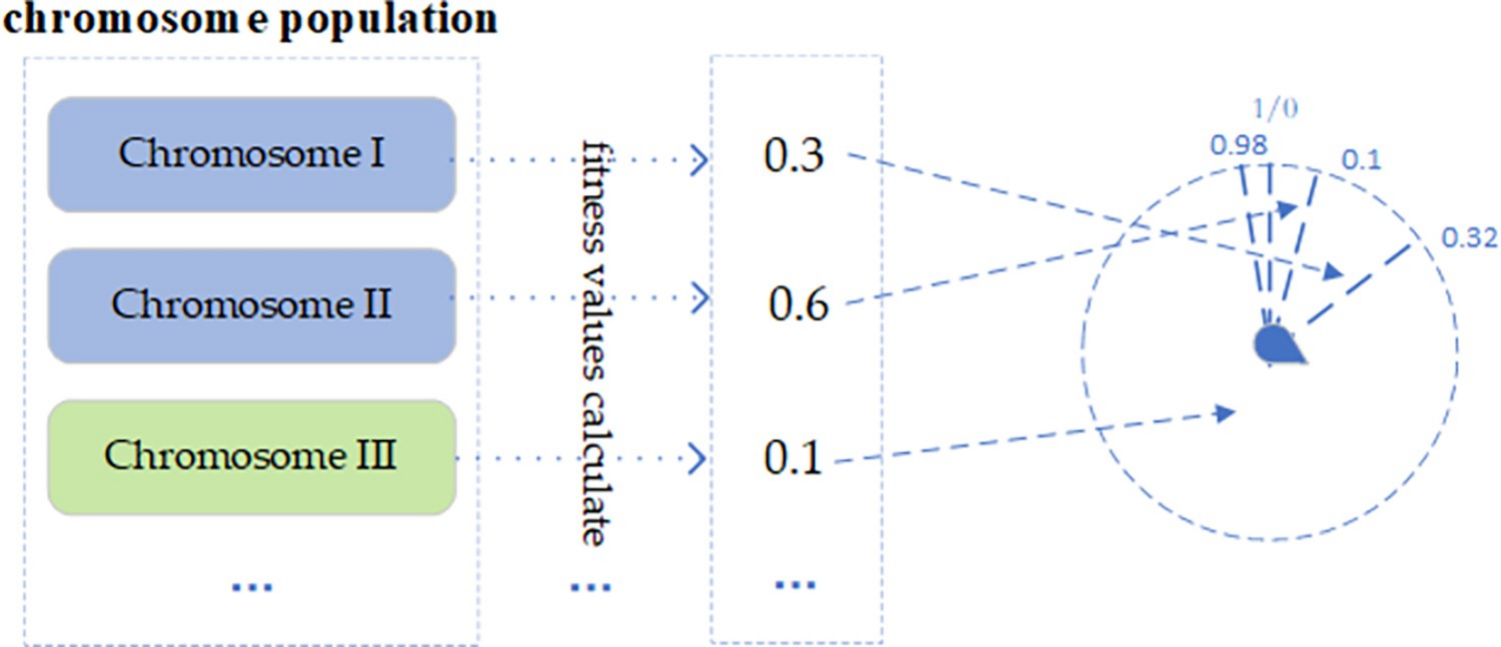
Process of chromosome selection.

The selection algorithm proceeds as follows and is illustrated in Fig 5:

Compute the fitness value for each chromosome within the population. Using the inverse of these values, construct a simulated roulette wheel.

ii)Generate a random selection probability. If this probability falls within the interval of a particular chromosome on the wheel, that chromosome is chosen.

Assume that the fitness of chromosomes I, II, and III are 0.3,0.6, and 0.1, respectively. Then, calculate the ratio of their reciprocal to the sum of all and they are about 22%,10%, and 66%. We add them in turn to get the value on the roulette wheel and finally, according to the random number 0.48 generated by the roulette pointer, we select chromosome III.

The crossover operation is invoked when a randomly generated number, pCross, exceeds the current crossover rate, which is dynamic and varies across generations. At the onset of the algorithm, a high crossover rate is essential to rapidly identify feasible solutions within the solution space. As the algorithm progresses, this rate is marginally reduced to refine feasible solutions and preserve superior chromosome segments. This dynamic crossover rate is encapsulated by Eq (5).


$$-\frac{{\left(currentIterateNum-2\right)}^{2}}{{totalIterateNum}^{2}}*crossRate+crossRate$$ (5)

Where currentIterateNum, totalIterateNum, and crossRate represent the current iteration round, total iteration rounds, and initial crossover rate, respectively.

For the crossover operation, two chromosomes are selected via the aforementioned selection algorithm. Subsequently, information on congruent loci in the genes of these chromosomes is exchanged.Crossover Process:

1. If pCross exceeds the current crossover rate, proceed; else, initiate mutation.
2. Generate crossover points randomly.
3. Via the selection algorithm, two chromosomes are chosen. The prior, central, and posterior domains at the identified crossover sites undergo an exchange of information.
4. Assess the viability of the post-crossover chromosome genes. If they’re viable, finalize the crossover and update the population. Otherwise, revert to the pre-crossover state.

Mutation process ensues post-crossover, triggered exclusively when a randomly generated number pChange surpasses the present mutation rate. This rate is dynamic, initiated at the start of the algorithm and then reducing non-linearly over time. This fluctuation is characterized by Eq (6).


$$-\frac{{\left(currentIterateNum-2\right)}^{2}}{{totalIterateNum}^{2}}*mutationRate+mutationRate$$ (6)

Where currentIterateNum, totalIterateNum, and changeRate represent the current iteration, total iterations, and initial mutation rate, respectively.

In our study, we employ single-point mutation, implying that a single locus is arbitrarily chosen for informational modification to enact the mutation. The procedure is:

1. If pChange is above the ongoing variation rate, proceed; else, halt the mutation.
2. Arbitrarily pinpoint mutation sites.
3. Utilizing the selection algorithm, mutations are induced in the prior, central, and posterior domains of the chosen chromosomal mutation site. Specifically, the prior domain undergoes random feature selection from the current discrete feature set, storing its index. Meanwhile, the central and posterior domains select values from the {0,1} set.
4. The viability of the mutated chromosome genes is assessed. If deemed viable, the mutation is finalized and the population data updated. If not, revert to the pre-mutation state.

#### Renewal iterations of chromosome populations

The iterative update is a cornerstone of the algorithm’s control logic. Its purpose is to ensure the algorithm consistently converges towards an optimal chromosome. To facilitate this, the most favorable chromosome from the preceding generation is retained within the current generation’s population. This strategic retention either promotes convergence towards that particular chromosome or incentivizes the search for a superior one. If the entire chromosome population of the current generation hasn’t undergone an update, the selection algorithm is invoked. This identifies a chromosome from the preceding generation’s population, subjecting it to crossover and mutation operations when corresponding rates are met. This continues until the entire current chromosome population has been iteratively updated. The process of renewal iteration is delineated below:

1. Increment the iteration count.
2. For each chromosome in the prior generation’s population:
  - Execute selection, crossover, and mutation operations.
  - Assess if the fitness value of the newly formed chromosome surpasses the minimum fitness value of the preceding generation’s chromosomes. If superior, retain the new chromosome. Otherwise, maintain the original.
3. Evaluate if the fitness value of the new chromosome exceeds the minimum fitness value from the prior generation. If so, keep the new chromosome; if not, revert to the original.
4. Construct a new generation of chromosome populations.

## Results and discussion

Experiments detailed herein were conducted on a system featuring Windows 10, an Intel® Core™ i5-7200U CPU (2.50GHz to 2.70GHz), and 8GB of RAM, with implementations in Python. In order to verify the effectiveness of the well-conceived gene coding in spatial storage, two datasets were employed additionally: the nursery dataset and breast-cancer dataset sourced from the University of California’s UCI Machine Learning Repository. Pertinent characteristics of both datasets can be found in Tables 4 and 5.

**Table 4:** Nursery dataset related notes.

Description items	Parameter Value
Number of data bars	12960
Number of attributes	9
Attribute 1—parents	usual, pretentious, great_pret
Attribute 2 -nursery	proper, less_proper, improper, critical, very_crit
Attribute 3 -family	complete, completed, incomplete, foster
Attribute 4—child	1, 2, 3, more
Attribute 5—housing	convenient, less_conv, critical
Attribute 6—finance	convenient, inconv
Attribute 7—society	non-prob, slightly_prob, problematic
Attribute 8—health	recommended, priority, not_recom
Attribute 9—recommended results	not_recom, recommend, very_recom, priority, spec_prior

**Table 5:** Breast-cancer dataset related notes.

Description items	Parameter Value
Number of data bars	286
Number of attributes	10
Attribute 1—class	no-recurrence-events, recurrence-events
Attribute 2 -age	10–19, 20–29, 30–39, 40–49, 50–59, 60–69,70–79,80–89,90–99
Attribute 3 -menopause	>40, <40, premeno
Attribute 4—tumor-size	0–4, 5–9, 10–14, 15–19, 20–24, 25–29, 30–34, 35–39, 40–44, 45–49, 50–54, 55–59
Attribute 5—inv-nodes	0–2, 3–5, 6–8, 9–11, 12–14, 15–17, 18–20, 21–23, 24–26, 27–29, 30–32, 33–35, 36–39
Attribute 6—node-caps	yes, no
Attribute 7—deg-malig	1, 2, 3
Attribute 8—breast	left, right
Attribute 9—breast-quad	left-up, left-low, right-up, right-low, central
Attribute 10—irradiat	Yes, no

Nursery database was derived from a hierarchical decision model originally developed to rank applications for nursery schools. It was used during several years in 1980’s when there was excessive enrollment to these schools in Ljubljana, Slovenia, and the rejected applications frequently needed an objective explanation. This dataset has a total of 12,960 records, each of which contains a total of 9 attributes, each with a different attribute value. The first eight attribute values have some correlation with the last attribute value.

Breast-cancer dataset was obtained from the University Medical Centre, Institute of Oncology, Ljubljana, Yugoslavia. This dataset has a total of 286 records, each of which contains a total of 10 attributes, each with a different attribute value. The first nine attribute values have some correlation with the last attribute value.

Our proposed DGAARM exhibits exemplary optimization concerning chromosome gene coding space storage, rule mining quantity, algorithm convergence, and rule quality. These findings offer invaluable insights for scholars exploring air quality determinants and furnish robust technological support for mining association rules in diverse application domains. Comprehensive comparative results and discussions are elucidated in subsequent sections.

### Comparison of chromosomal gene coding space storage

**Fig 6:**
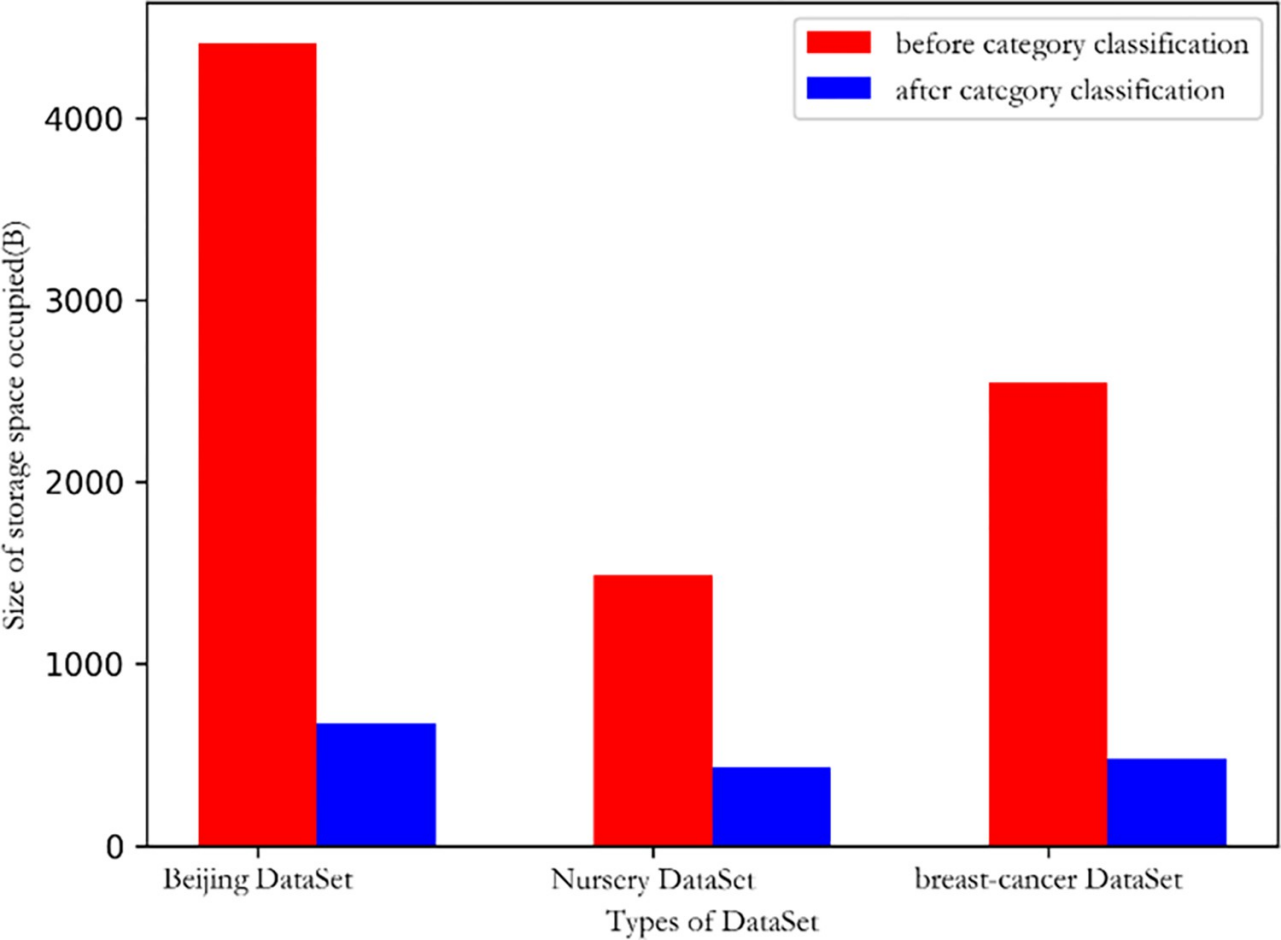
Comparison of chromosomal gene coding space storage before and after data set category classification.

A salient distinction in chromosomal genetic code space storage stems from the categorization status of dataset items. Grouping items permits the consolidation of non-sequitur related or similar-category items within a shared storage, negating the necessity for discrete storage spaces. Such a coding paradigm effectively diminishes the redundancy between analogous items, optimizing space usage, which delineated in Fig 6. Since the specific categories under the features of the Beijing dataset are more than those of the other two datasets, the space can be reduced more significantly.

#### Rule number mining comparison

**Fig 7:**
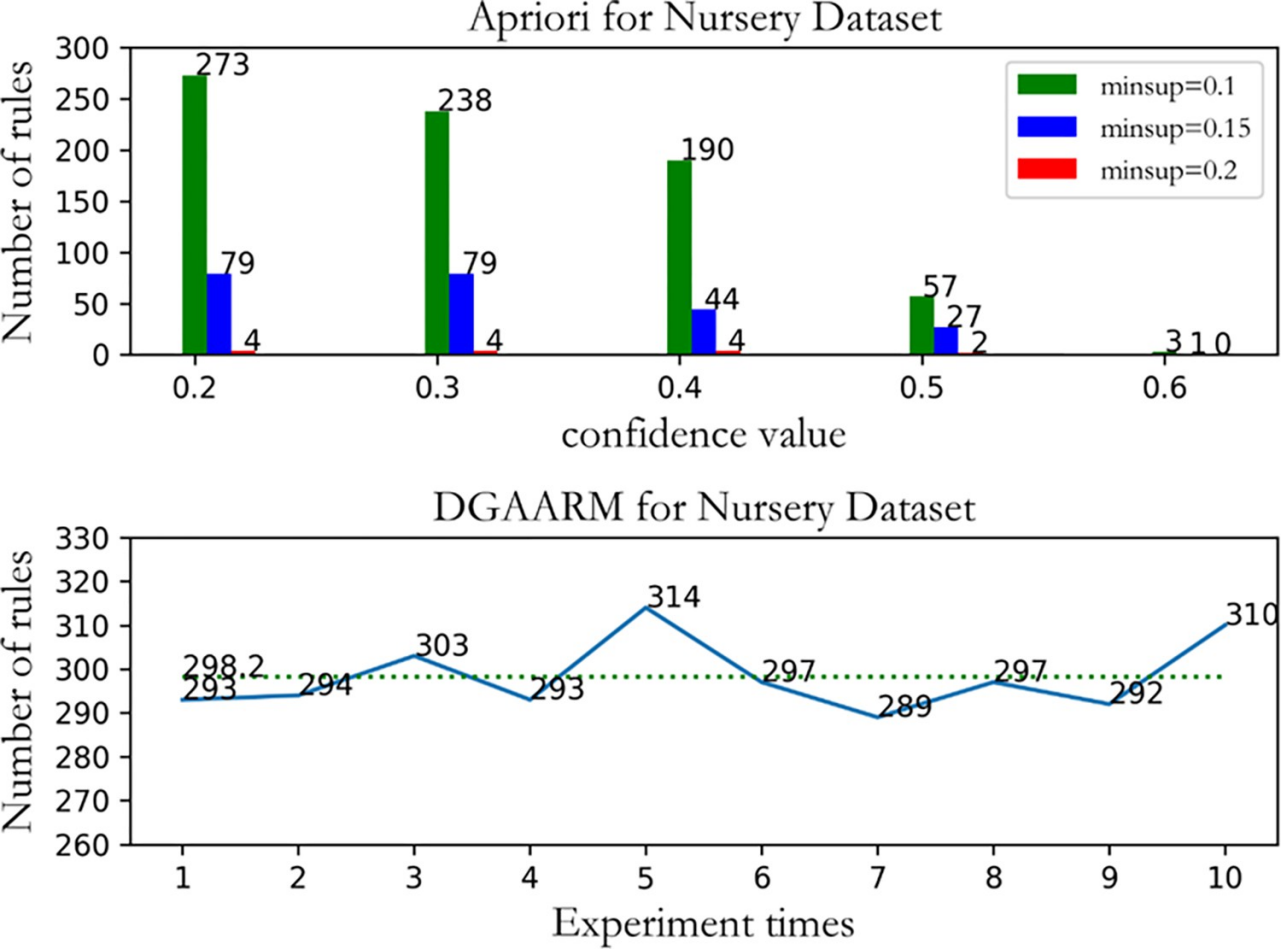
Comparison of the number of Apriori and DGAARM rule mining on the nursery dataset.

In the aspect of rule number mining, we find that because traditional association rule algorithms are affected by manual support settings, the number of rules mined under different support thresholds is different. When the support level is low, the number of rules that can be mined is large. As the support level increases, the number of rules that can be mined is significantly reduced, because the manual support settings filter out a large number of item sets. As we can see, when applying Apriori to Nursery data sets, a large number of rules can be mined when minimum support is set to 0.1 and minimum confidence is set to 0.2, while rules are no longer mined when minimum support is set to 0.2 and minimum confidence is set to 0.6. However, when DGAARM was applied to 10 repeated experiments on the Nursery dataset, it was found that by eliminating the step of setting the threshold parameter, it consistently looked for high-quality rules out of about 298 rules, as shown in Fig 7. Table 6 lists the rules on some Nursery datasets that are mined in part by DGAARM. For example, the family in society is problematic, then his financial aspect is inconvenient or families recommended for admission are financially convenient. We can derive the rules based on the weight of the evaluation, rather than filtering some item set by support thresholds

**Table 6:** Extracting association rules on the Nursery dataset using DGAARM with different weights of support, confidence, and lift.

Rules	Support weight	Confidence weight	Lift weight	Interest level
{society-problem} = >{finance-inconvenient}	0.1	0.9	0	0.46
{result-recommended} = >{finance-convenient}	0.1	0.9	0	0.90
{family-foster care, health-healthy} = >{result-preferred}	0.2	0.8	0	0.43
{finance-convenient} = >{family-complete}	0.3	0.4	0.3	0.28
{finance-convenient} = >{result-preferred,child-3,family-incomplete,nursery-very critical}	0.4	0.5	0.1	0.50
{child-3, parents-ordinary} = > {finance-inconvenient}	0.4	0.5	0.1	0.32
{society-minor problem} = >{housing-inconvenient}	0.5	0.4	0.1	0.22

**Fig 8:**
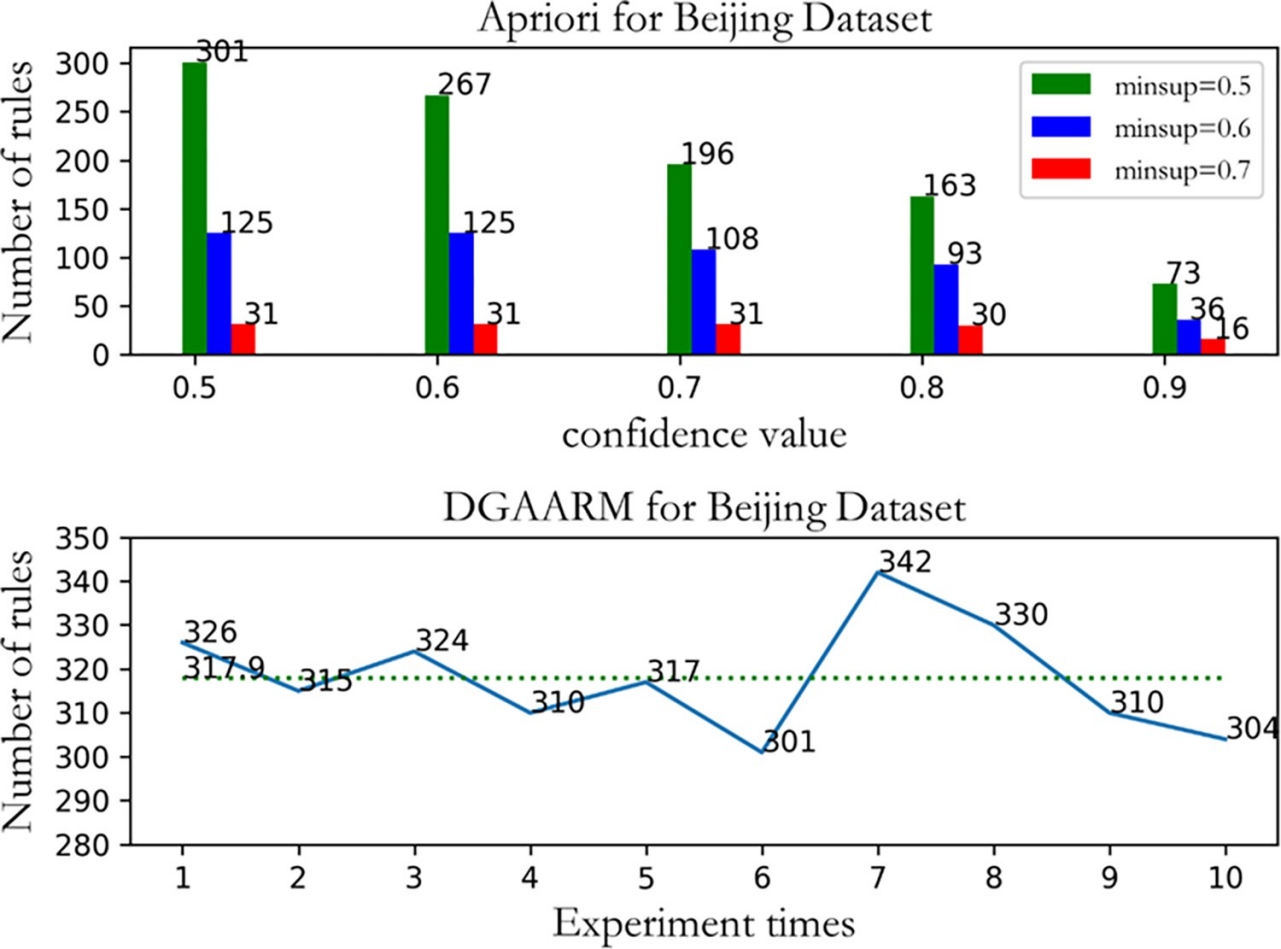
Comparison of the number of Apriori and DGAARM rule mining on the Beijing dataset.

Similarly, we can see from Fig 8 that when Apriori is applied to the Beijing dataset, a large number of rules can be mined when the minimum support is set to 0.5 and the minimum confidence is set to 0.5, while the minimum rules are mined when the minimum support is set to 0.7 and the minimum confidence is set to 0.9. However, when DGAARM was applied to 10 repeated experiments on the Beijing dataset, it could consistently look for high-quality rules from about 318 rules.

### Comparison between DGAARM and other rule mining methods

This experiment provides a comprehensive comparison of the DGAARM algorithm with a range of traditional association rule mining algorithms including Apriori, FP-Growth, and Eclat. In addition, we also compare rule mining algorithms that integrate swarm intelligence, such as Particle Swarm Association Rule Mining (PSOARM), Multi-objective Particle Swarm Association Rule Mining (MOPSOARM), Whale Association Rule Mining (WOAARM), Differential Evolution Association Rule Mining (DEARM), as well as Animal Dynamic Migration Association Rule Mining (ADMOARM).

**Fig 9:**
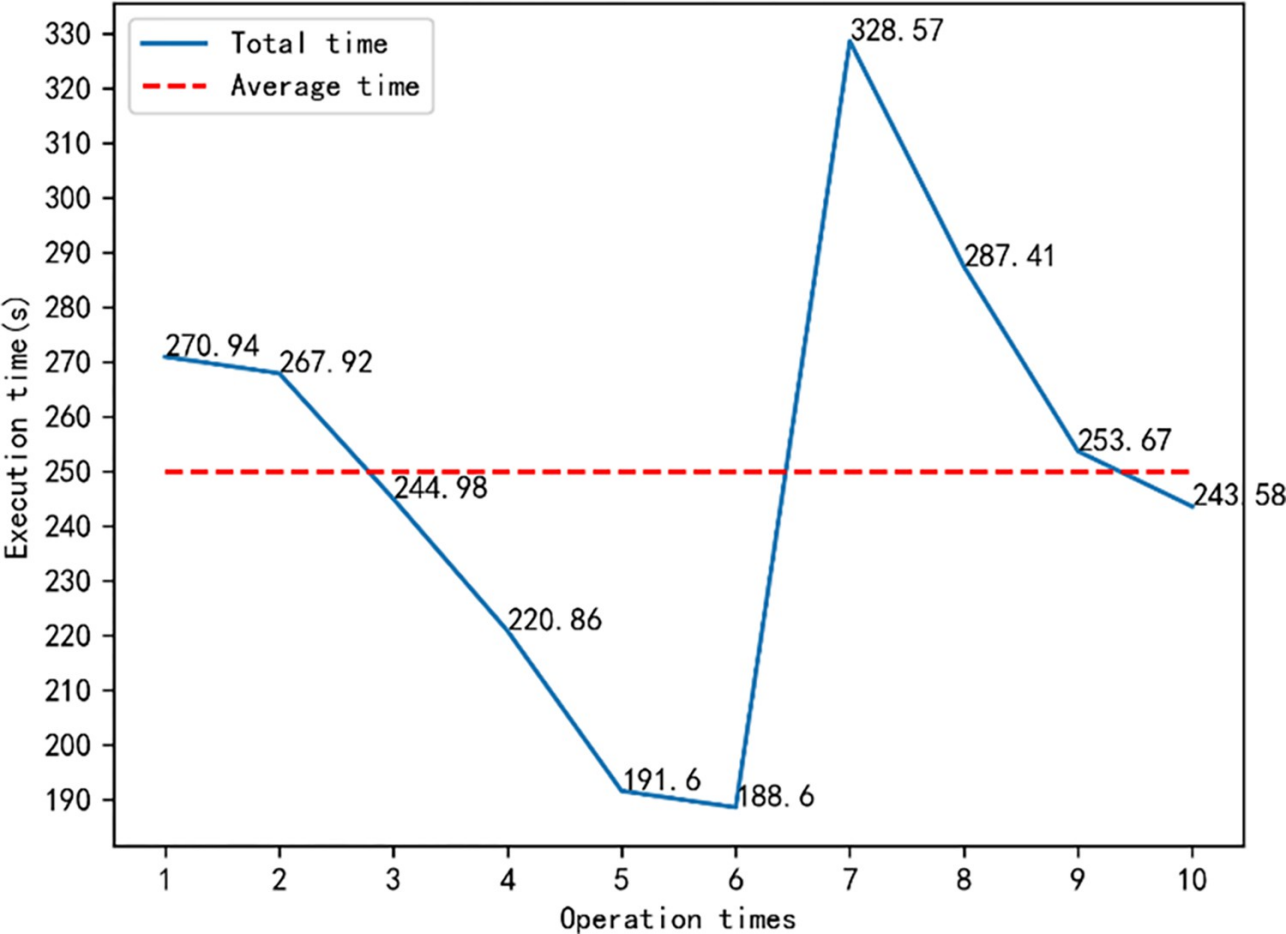
Single and average running time of the DGAARM algorithm for 10 times.

Key performance indicators employed in this comparison include rule mining time consumption, with pertinent results presented in Table 7. During the testing phase, algorithmic parameters such as support, confidence, and lift were calibrated at weights of 0.3, 0.6, and 0.1 respectively. Each algorithm was executed ten times, with individual run times recorded and visually represented in Fig 9.

**Table 7:** Comparison of running time between DGAARM algorithm and Apriori and other 8 algorithms on the Beijing data set.

Methods	Time (s)
Apriori	156.322
FP-Growth	148.804
Eclat	211.027
PSOARM	313.510
MOPSOARM	320.008
WOAARM	352.201
DEARM	302.129
ADMOARM	270.301
**DGAARM (ours)**	**249.813**

**Fig 10:**
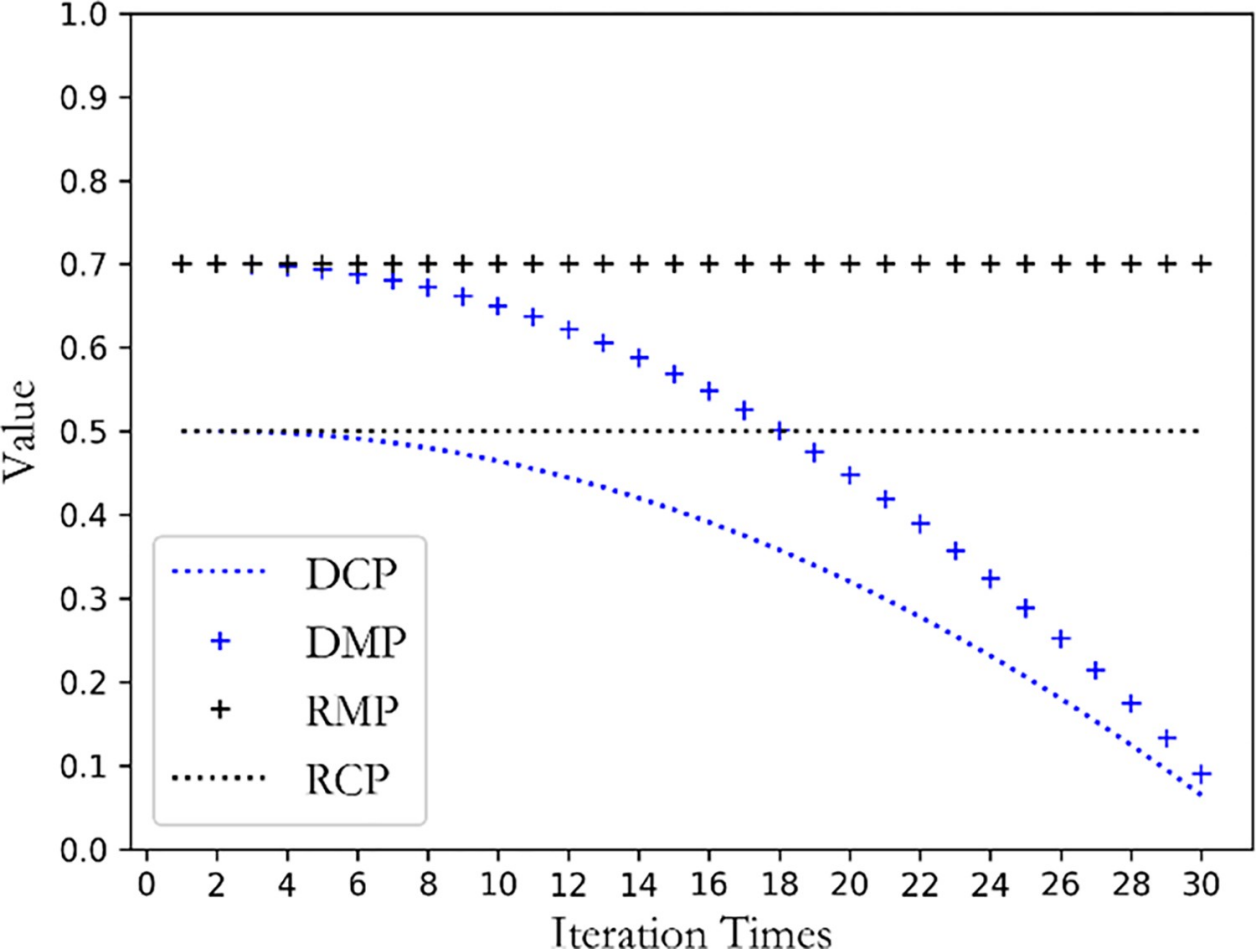
Comparison of raw and dynamic crossover/mutation rate change process.

Insights derived from Table 7 indicate that DGAARM’s runtime marginally surpasses that of conventional rule mining algorithms. This variance can be attributed to the latter’s approach of setting minimum thresholds and consequently filtering specific items, optimizing run times. Remarkably, when juxtaposed with swarm intelligence-optimized rule mining algorithms, DGAARM boasts the most efficient execution time. Such efficiency stems from its superior coding methodology combined with a dynamic search strategy, specifically its dynamic crossover and mutation rates, detailed further in Fig 10.

**Fig 11:**
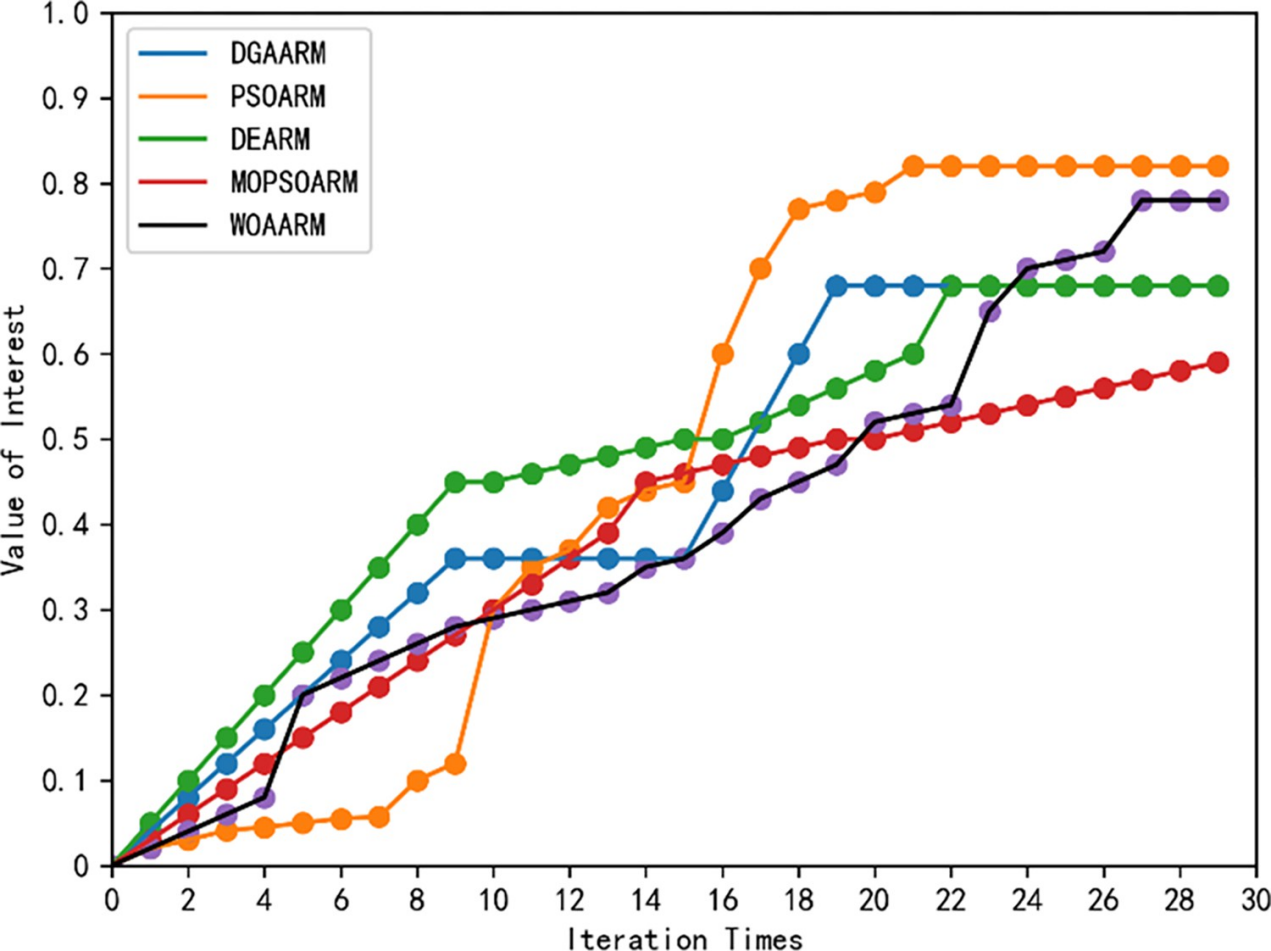
Comparison of convergence of the DGAARM algorithm with the other five optimization algorithms after 30 iterations.

To illustrate the advantages of DGAARM in terms of convergence performance, we set the number of iterations of each algorithm to 30 and record the interesting-ness value of the optimal rule in each iteration. Inspection of Fig 11 reveals that the algorithm’s performance convergence aligns well with theoretical expectations. Specifically, during the initial phases, DGAARM rapidly identifies rules of high interest when both the crossover and mutation rates are elevated. As these rates stabilize at lower values in subsequent stages, the algorithm converges the interest values of the rules to a stable equilibrium. Notably, due to its dynamic search strategy, DGAARM achieves a more efficient convergence time compared to other algorithms such as PSOARM, MOPSOARM, WOAARM, and DEARM.

#### Rule quality analysis

DGAARM is engineered to discern rules of interest that yield superior quality. In this paper, a unique approach is put forward wherein varying weight values for support, confidence, and lift serve as a fitness function, thus enabling the extraction of rules with superior function values. Table 8 lists some of the rules mined using DGAARM. When focusing on rule confidence, for instance, we can get the rule {temperature_warm} = > {barometric pressure_low}. This is coherent with the environmental reality that balmy temperatures correlate with lower barometric pressures.

**Table 8:** Extracting air quality influencing factor association rules using DGAARM in Beijing meteorological dataset.

Rules	Support weight	Confidence weight	Lift weight	Interest level
{O_3__II, Temperature_Warm} = >{SO_2__I, Rainfall_R0}	0.1	0.9	0	0.9
{Temperature_Warm} = >{Barometric pressure_Low pressure}	0.1	0.9	0	0.79
{AQI-5, Wind power_Force 3 wind, Wind direction_Northeast} = >{Barometric pressure_High pressure, Relative humidity_Normal, Rainfall_R0}	0.1	0.9	0	0.20
{Relative humidity_Humidity, Wind direction_Northeast} = >{Rainfall_R1, Wind power_Force 3 wind}	0.2	0.2	0.6	0.60
{AQI-1} = >{PM10_I, Temperature_Warm, Relative humidity_Humidity}	0.2	0.2	0.6	0.60
{AQI-5} = >{CO_II, Weather condition_Overcast, Temperature_Lightly cold, Wind power_Force 3 wind}	0.2	0.2	0.6	0.60
{Barometric pressure_High pressure} = >{Rainfall_R0}	0.3	0.3	0.4	0.62
{AQI-1, Barometric pressure_High pressure} = >{O_3__I}	0.3	0.3	0.4	0.44
{NO_2__III, PM10_II, Barometric pressure_High pressure, Temperature_Slightly cold} = >{SO_2__I}	0.4	0.4	0.2	0.36
{Barometric pressure_High pressure} = >{PM10_I,PM2_5_I}	0.4	0.4	0.2	0.30
{O_3__I} = >{SO_2__I}	0.5	0.5	0	0.87
{CO_I, Relative humidity_Normal, Wind power_Light breeze} = >{NO_2__II}	0.5	0.5	0	0.32
{Weather condition_Sunny, Barometric pressure_Low pressure, Temperature_Warm, Wind power_Force 3 wind} = >{SO_2__I}	0.7	0.2	0.1	0.20
{CO_I, SO_2__I, Barometric pressure_Low pressure} = >{O_3__II}	0.7	0.2	0.1	0.20
{AQI-1, PM2_5_I} = >{Barometric pressure_High pressure}	0.8	0.1	0.1	0.25
{NO_2__V, Relative humidity_Humidity} = >{CO_III, SO_2__III, Barometric pressure_High pressure}	0.8	0.1	0.1	0.20

Similarly, when emphasizing rule lift, the algorithm unveils the rule {AQI-1} = > {PM10_I, temperature_mild, relative_humidity}. This illustrates the scenario when excellent air quality is accompanied by minimal PM10 concentrations, mild temperatures, and relatively humid conditions.

In instances where support is the emphasis, akin to classical algorithms such as Apriori, it is possible to derive the rule {O_3__I} = >{SO_2__I}. This suggests that both ozone and SO_2_ concentrations are classified at rank one. In addition, Table 9 lists six items with low support in the Beijing data set. When the traditional method such as Apriori is used to set the support level to 0.2, it is impossible to mine the rules, but these rules can be found in Table 8, such as {O3_II, Temperature_Warm} = > {SO2_I, Rainfall_R0}, {AQI_V} = > {CO_II, Weather condition_Overcast, Temperature_Lightly cold, Wind power_Force3 wind}, etc.

**Table 9:** Six low support items in Beijing meteorological dataset.

Item	support
Temperature_Lightly cold	0.1113
O_3__II	0.1126
Temperature_Slightly cold	0.1230
Wind power_Force 3 wind	0.1539
NO_2__III	0.1686
Relative humidity_Humidity	0.1766

It is critical, however, to note that excessive concentration on the aspect of support could lead to the extraction of rules with markedly reduced interest levels, potentially yielding results of low relevance to the researcher.

This resonates with the issue that conventional association rules might fail to identify pertinent rules when the support level is high. In such scenarios, redirecting focus onto other evaluative metrics via the DGAARM algorithm could prove highly beneficial.

### Statistical analysis

In order to better show that our algorithm is not limited by the minimum support threshold and can mine the rules with low support, a statistical analysis is carried out. The steps are as follows: First we establish the null hypothesis, which is the argument described above, and by rejecting this null hypothesis we can statistically prove our validity. Secondly, we conducted random statistics on the support degree of rules mined by DGAARM and traditional algorithm Apriori on the Beijing dataset, and then conducted Student’s t test, which is a statistical method used to measure the deviation degree of observed values from expected values. Table 10 shows the test results. The value for μ, standard deviation (s), t-value (t) and p-value of each algorithm are obtained from the t-test, where μ represents the average support degree of the mined rule, and s represents its standard deviation of the rules mined. The standard deviation is calculated by Eq (7), the t-value is calculated by Eq (8), and n1 and n2 are the number of random sample rules, which are set as 10.


$$S=\sqrt{\frac{\sum_{i=1}^{n}{\left(x_{i}-\bar{x}\right)}^{2}}{n}}$$ (7)

$$t=\frac{{\bar{X}}_{1}-{\bar{X}}_{2}}{\sqrt{\frac{(n_{1}-1)s_{1}^{2}+(n_{2}-1)s_{2}^{2}}{n_{1}+n_{2}-2}(\frac{1}{n_{1}}+\frac{1}{n_{2}})}}$$ (8)

**Table 10:** Student’s t test of DGAARM and Apriori.

Beijing(support = 0.3)				
	μ	s	t	p-Value
DGAARM	0.265	0.0193	3.326	0.00375
Apriori	0.561	0.0522		

The p-value can be calculated by subtracting the normal distribution value of t from 1. If the p-value is lower than a given significance level α, the established null hypothesis can be rejected. In this experiment, we set the significance level α as 0.05 (5%). In Table 10, a significance level of p-value less than 0.05 can be observed. Therefore, we can determine that there is a statistically significant difference between DGAARM and the traditional rule Apriori in mining rules that are exempt from support threshold setting. In other words, we can mine rules that are exempt from support thresholds.

## Conclusions

With the gradual deepening of the process of economic globalization, environmental problems brought by rapid economic development have attracted more and more attention. Good air quality is crucial to people’s physical and mental health and social activities. In addition, the current global climate change is accelerating, and extreme weather conditions are also having a huge impact on air quality. Under the double influence, it is of great significance to explore the correlation between air quality and meteorological sources. This manuscript innovatively proposes DGAARM based on traditional genetic algorithm and association rule mining technology, and applies it to air pollution correlation analysis, which can effectively reveal the correlation between air quality and various factors at different levels. Key findings from the experimental outcomes highlight:

1. The design of a novel gene coding strategy that is rooted in a single locus and capable of carrying multiple information chromosomes reduced the rule space storage consumption by 50%.
2. The dynamic crossover and mutation rate are proposed in the process of optimal search, which makes the algorithm have strong global search ability in the initial execution, and transition to fast convergence in the subsequent algorithm iteration. Based on it and the special coding strategy above, we reduced about 20% in terms of search time compared with some heuristic algorithm, while improving convergence.
3. The implementation of the algorithm’s new evaluation index is not limited by the threshold of support and confidence, and can stably mine the association rules, whose interest level can be up to 90% between frequent and infrequent items in the object database.
4. DGAARM can complete air quality impact factor mining after preprocessing complex Beijing data sets including discrete and continuous data. Future research will consider adapting more different types of air quality data in the future, as well as integrating clustering technology into the data preprocessing stage to increase mining at different feature levels.
